# Simultaneous Thoracoscopic Approach in a Patient with Long-Standing Persistent Atrial Fibrillation and Primary Lung Cancer: the First Described Case

**DOI:** 10.21470/1678-9741-2019-0143

**Published:** 2020

**Authors:** Aleksandr Zotov, Sergei Vachev, Daniil Borisov, Aleksandr Troitskiy, Robert Khabazov

**Affiliations:** 1 Department of Cardiac Surgery, Federal Research and Clinical Centre, Moscow, Russian Federation.

**Keywords:** Lung Cancer Surgery, Atrial Fibrillation (AF), Flutter, Thoracoscopy/VATS, Arrhythmia Therapy (Incl Ablation, Surgery, Drugs)

## Abstract

Cardiac rhythm disorders are common in many patients with cancer. The management of synchronous long-standing persistent atrial fibrillation and pulmonary lesions remains a serious surgical dilemma due to the lack of clinical data and surgical guidelines. To the best of our knowledge, this is the first described case of simultaneous thoracoscopic pulmonary segmentectomy and left atrial posterior wall and pulmonary vein isolation combined with left atrial appendage resection in a patient with early-stage primary lung cancer and long-standing persistent atrial fibrillation.

**Table:** 

Abbreviations, acronyms & symbols	
CT	= Computed tomography
ICS	= Intercostal space
LSPAF	= Long-standing persistent atrial fibrillation

## INTRODUCTION

Coexisting malignancies and long-standing persistent atrial fibrillation (LSPAF) requiring treatment with radiofrequency ablation are not rare in clinical practice. We herein report a minimally invasive approach involving a combination of thoracoscopic left atrial posterior wall and pulmonary vein isolation with left atrial appendage resection and thoracoscopic segmental resection of right S6 for simultaneous treatment of atrial fibrillation and lung cancer. As far as we know, this is the first described case of simultaneous thoracoscopic approach to treat a patient with LSPAF and primary lung cancer.

## CASE REPORT

A 65-year-old woman with a history of LSPAF (> 15 years) was referred for thoracoscopic left atrial posterior wall and pulmonary vein isolation combined with left atrial appendage resection. After being treated with amiodarone, digoxin, and beta-blockers for several years, her electrocardiogram remained abnormal. Electrical cardioversions eight and six years before the admission were unsuccessful. Anticoagulant (Rivaroxaban) use was stopped after intestinal bleeding two years before the surgery.

**Fig. 1:**
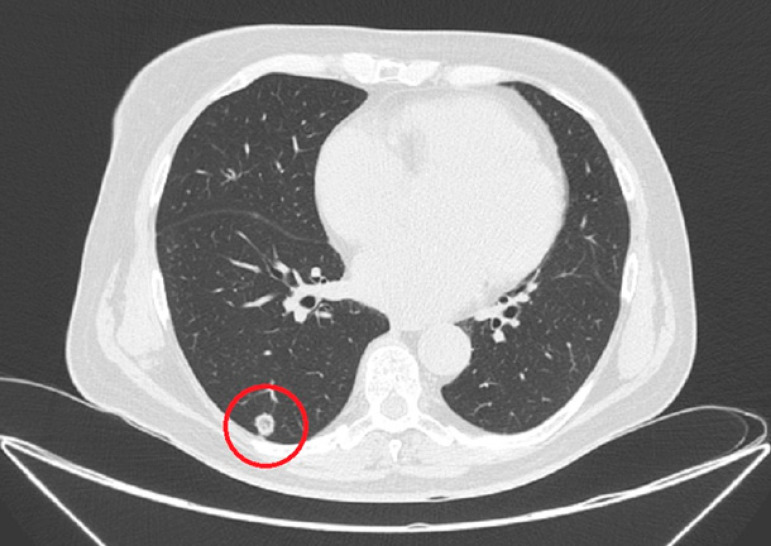
Computed tomography scan of the mass.

Brain natriuretic peptide on admission was elevated to 1621 pg/mL, while troponin I was negative. Echocardiography showed moderate mitral and tricuspid regurgitation, pulmonary hypertension (mean arterial pressure 41 mmHg at rest), moderate impairment of left ventricular systolic motion, a left ventricular ejection fraction of 50%, and atrial dilatation, with a left atrial diameter of 43 mm. Coronary angiography showed arteries without any sign of obstructive atherosclerosis. Preoperative chest computed tomography (CT) is a part of the protocol for patients undergoing video-assisted thoracoscopic procedures at our institution and it revealed a mass in the superior segment (S6) of the inferior lobe of the right lung (Figure 1).

In order to follow the institutional protocol for cancer patients and minimize the operative trauma, we performed a totally thoracoscopic resection of the lung lesion and left atrial posterior wall and pulmonary vein isolation. Catheter ablation of atrial fibrillation was not performed because of its controversial efficacy in patients with LSPAF^[1-3]^.

The operation was carried out under general anesthesia with single lung ventilation. The patient was positioned supine with 45° rotation toward the left decubitus position. External pads for emergency defibrillation were placed on the chest wall. The surgeon stood in the right side of the patient to perform segmentectomy first.

Three ports were made, including a 1-cm thoracoscopic port in the 6^th^ intercostal space (ICS) on the scapular line, a 2-cm incision on the middle axillary line in the 4^th^ ICS, and a 2-cm incision in the 5^th^ ICS on the anterior axillary line for resection of the superior segment of the right inferior pulmonary lobe using a thoracoscopic linear stapler (EndoGIA, Medtronic). The tumor had dark grey appearance and was completely included in the S6. The diagnosis of the lesion was confirmed by intraoperative frozen section.

After that, the patient was positioned in supine position on the table to perform left atrial posterior wall and pulmonary vein isolation combined with left atrial appendage resection (also known as GALAXY procedure^[4]^). At our institution, the modified GALAXY procedure is routinely used. We increased the number of ablations to 20 for each side (the total number of ablations is 40) with frequent position changing of the device and change of device angulation after 10 ablations (technique described below).

Briefly, the procedure is performed through three thoracic ports with pericardial reflection and sequential single lung ventilation. Soft grey and blue rubber catheters are passed behind the left atrium, above and below the superior and inferior pulmonary veins, respectively, to guide the device advancement while avoiding trauma.

**Fig. 2:**
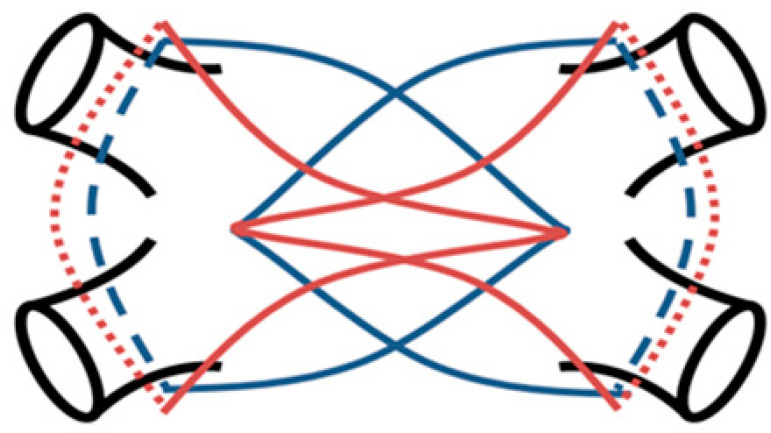
Scheme of left atrial posterior wall and pulmonary vein ablation lines.

The curved device is firstly inserted into the pericardial cavity from the left side, guided by the soft rubber catheters, up to the patient's spine. Once appropriately positioned, it is clamped over the left half of the left atrium. Lesions are applied guided by impedance drop to confirm transmurality. After each application, the device is adjusted a little to get overlapping lesions. The process is repeated on the right side to obtain isolation of the pulmonary veins as well as a large portion of the left atrial posterior wall (Figure 2).

The final portion of the procedure is removal of the left atrial appendage with use of a thoracoscopic stapling device (EndoGIA, Medtronic).

After the surgery, the patient was transferred to the intensive care unit and recovered well. Sinus rhythm was restored 12 hours later, after amiodarone infusion and electrical cardioversion.

A perioperative red blood cell transfusion was not necessary. The patient’s postoperative mechanical ventilation time was 12 hours. Postoperative transthoracic echocardiography showed normal ejection fraction and heart chamber. Pathologic examination of the lung lesion showed a well differentiated primary pulmonary mucinous adenocarcinoma. Patient was discharged on the 6^th^ postoperative day.

Three-month, 6-month, and 1-year follow-ups (24-hour Holter monitoring) revealed no sign of recurrence of atrial fibrillation or other supraventricular arrhythmia.

Postoperative therapy included amiodarone, warfarin (canceled after three months), and bisoprolol (the only medicine continued in this patient after the 3-month follow-up).

Chest CT at 3-month and 1-year follow-ups showed no sign of tumor recurrence and good expansion of the residual right lower lobe with no consolidation. No chemotherapy or radiotherapy was needed.

## DISCUSSION

Thoracoscopic pulmonary segmentectomy is feasible and safe in selected patients with small tumors^[5]^. When compared with thoracoscopic lobectomy, thoracoscopic segmentectomy had similar rates of morbidity, recurrence, and survival. When compared with open segmentectomy, thoracoscopic segmentectomy was found to have equivalent oncologic results, with shorter hospital length of stay, reduced rates of morbidity, and lower cost. It was also found that thoracoscopic segmentectomy results in greater preservation of lung function and exercise capacity than the thoracoscopic lobectomy^[5]^.

Concomitant cardiac rhythm disorders and lung cancer are not uncommon and may pose a serious surgical dilemma. No formal consensus regarding the optimal treatment of patients with early-stage lung cancer and atrial fibrillation, whether by contemporary or staged surgical treatment, has been reached. Cancer resection followed by atrial fibrillation treatment may increase the perioperative and intraoperative cardiogenic risks to the patient, while atrial fibrillation radiofrequency ablation followed by cancer resection is associated with a risk of intraoperative carcinoma metastasis. Combined one-stage cancer resection and Cox-Maze IV procedure may be associated with a high risk of surgical trauma to the patient. A one-stage, minimally invasive combined surgical procedure provides a new option for these patients. The advantages of a one-stage approach include use of fewer anesthetic agents, less operative stress and pain, a shorter hospital stay, lower therapeutic cost, and less delay of tumor treatment. Totally thoracoscopic left atrial posterior wall and pulmonary vein isolation combined with left atrial appendage resection has become a routine operation in clinical practice. In the present case, the combination of thoracoscopic left atrial posterior wall and pulmonary vein isolation and resection of the primary lung lesion had excellent short-term and long-term results.

**Table:** 

Author's roles & responsibilities	
AZ	Substantial contributions to the conception or design of the work; or the acquisition, analysis, or interpretation of data for the work; drafting the work or revising it critically for important intellectual content; final approval of the version to be published
SV	Substantial contributions to the conception or design of the work; or the acquisition, analysis, or interpretation of data for the work; drafting the work or revising it critically for important intellectual content; final approval of the version to be published
DB	Substantial contributions to the conception or design of the work; or the acquisition, analysis, or interpretation of data for the work; drafting the work or revising it critically for important intellectual content; final approval of the version to be published
AT	Substantial contributions to the conception or design of the work; or the acquisition, analysis, or interpretation of data for the work; drafting the work or revising it critically for important intellectual content; final approval of the version to be published
RK	Substantial contributions to the conception or design of the work; or the acquisition, analysis, or interpretation of data for the work; drafting the work or revising it critically for important intellectual content; final approval of the version to be published
